# Association of CD40 Gene Polymorphisms with Sporadic Breast Cancer in Chinese Han Women of Northeast China

**DOI:** 10.1371/journal.pone.0023762

**Published:** 2011-08-30

**Authors:** Chen Shuang, Li Dalin, Yuan Weiguang, Fu Zhenkun, Xu Fengyan, Pang Da, Dianjun Li

**Affiliations:** 1 Department of Immunology, Harbin Medical University, Harbin, China; 2 Department of Surgery, The Third Affiliated Hospital of Harbin Medical University, Harbin, China; 3 Institute of Cancer Prevention and Treatment, Harbin Medical University, Harbin, China; Beth Israel Deaconess Medical Center, United States of America

## Abstract

**Background:**

Breast cancer is a polygenetic disorder with a complex inheritance pattern. Single nucleotide polymorphisms (SNPs), the most common genetic variations, influence not only phenotypic traits, but also interindividual predisposition to disease, treatment outcomes with drugs and disease prognosis. The co-stimulatory molecule CD40 plays a prominent role in immune regulation and homeostasis. Accumulating evidence suggests that CD40 contributes to the pathogenesis of cancer. Here, we set out to test the association between polymorphisms in the CD40 gene and breast carcinogenesis and tumor pathology.

**Methodology and Principal Findings:**

Four SNPs (rs1800686, rs1883832, rs4810485 and rs3765459) were genotyped by the polymerase chain reaction restriction fragment length polymorphism (PCR-RFLP) method in a case-control study including 591 breast cancer patients and 600 age-matched healthy controls. Differences in the genotypic distribution between breast cancer patients and healthy controls were analyzed by the Chi-square test for trends. Our preliminary data showed a statistically significant association between the four CD40 gene SNPs and sporadic breast cancer risk (additive P = 0.0223, 0.0012, 0.0013 and 0.0279, respectively). A strong association was also found using the dominant, recessive and homozygote comparison genetic models. In the clinical features analysis, significant associations were observed between CD40 SNPs and lymph node metastasis, human epidermal growth factor receptor 2 (C-erbB2), estrogen receptor (ER), progesterone receptor (PR) and tumor protein 53 (P53) statuses. In addition, our haplotype analysis indicated that the haplotype C_rs1883832_G_rs4810485_, which was located within the only linkage disequilibrium (LD) block identified, was a protective haplotype for breast cancer, whereas T_rs1883832_T_rs4810485_ increased the risk in the studied population, even after correcting the P value for multiple testing (P = 0.0337 and 0.0430, respectively).

**Conclusions and Significance:**

Our findings primarily show that CD40 gene polymorphisms contribute to sporadic breast cancer risk and have a significant association with clinicopathological features among Chinese Han women from the Heilongjiang Province.

## Introduction

Breast cancer is a major cause of cancer death among women worldwide. Recent research has suggested that variations in some immune regulatory genes drive interindividual differences in sporadic breast cancer susceptibility [1]. CD40 is a crucial member of the group of co-stimulating molecules, orchestrating both humoral and cell-mediated immune responses. The interaction between the CD40 protein and its ligand CD154 triggers a number of signaling events affecting antigen-presenting cell (APC) activity, T cell costimulation, and B cell growth and survival [2]. Moreover, CD40 is not only expressed on immune cells but is also present on endothelial cells and fibroblasts [3]–[6]. Nearly 75% of all epithelial malignancies examined to date may express a high level of CD40, indicating the complex functions of this molecule in human disease pathogenesis. CD40 is a member of the tumor necrosis superfamily and is located on chromosome 20q12-13.2. The CD40 pathway plays an important role in anti-tumor responses by promoting cytotoxic lymphocyte (CTL) responses and the differentiation of helper T cells into cells with the Th1 phenotype [7]. In recent years, some CD40 agonists have been demonstrated to be effective against human malignancies [8], [9]. Conversely, other studies have shown that CD40 may contribute to tumor growth and metastasis [10]. For instance, CD40 signaling in endothelial cells results in less apoptosis and increased proliferation, which may promote tumor growth through angiogenesis [11]–[13]. In addition, chemokines, such as IL-10 and VEGF, induced by the CD40-CD153 interaction on various cell types are all thought to have roles in malignant cell metastasis [7]. Currently, the mechanisms of the multifaceted roles of CD40 in cancer are still not completely understood.

Genetic polymorphism is a key element that affects the susceptibility to breast cancer. A recent report in a Turkish population study showed there is an association between the rs1883832 TT genotype and susceptibility to breast cancer [14]. Furthermore, some functional studies have reported that rs1883832 is associated with enhanced CD40 translational efficiency and influences the levels of CD40 molecules on the surfaces of B lymphocytes and dendritic cells and the level of circulating soluble CD40 [15], [16]. In addition, CD40 polymorphisms have roles in predicting infection- and autoimmunity-associated diseases, such as Grave's disease and coronary artery calcification [16], [17].

Based on these data, we speculated that SNPs in CD40 independently or synergistically influence the susceptibility to sporadic breast cancer. To investigate this hypothesis, a case-control study was conducted in a Chinese Han population from Heilongjiang Province, located in the northeastern part of China.

## Results

### Frequencies of genotypes and alleles

The distribution of the CD40 genotypes in our studied population is given in Table 1. To ensure quality control of the genotyping results, samples that failed to be genotyped in the first round of genotyping were run a second time. Samples for which genotyping data was successfully obtained from the second round were then repeated once, whereas samples for which the genotypes remained uninterpretable were classified as missing data. Furthermore, we randomly selected 10% of the samples to submit to direct sequencing, and the results were consistent with the PCR-RFLP results. In total, 1177 samples (98.82%) of the rs1800686 polymorphism, 1165 samples (97.82%) of the rs1883832 polymorphism, 1178 samples (98.91%) of the rs4810485 polymorphism and 1157 samples (97.15%) of the rs3765459 polymorphism were successfully tested in this study. For all of the genotyped SNPs, there was no deviation from Hardy-Weinberg equilibrium (P>0.05), and no minor allele frequency was less than 10%. Missing data accounted for less than 10% of all data.

**Table 1:** Genotyping of CD40 gene SNPs in breast cancer patients and controls.

Reference SNP ID	Geno -type	Frequency no. (%)		Additive P value	Dominant P value	Recessive P value	Homozygote comparison P value
		Patients	Controls				
rs1800686	GG	265(45.69%)	293(49.08%)	**0.0223**	0.2444	**0.0060**	**0.0071**
	AG	234(40.34%)	251(42.04%)				
	AA	81(13.97%)	53(8.88%)				
rs1883832	CC	213(36.66%)	274(46.92%)	**0.0012**	**0.0004**	0.8316	0.1438
	CT	297(51.12%)	241(41.27%)				
	TT	71(12.22%)	69(11.82%)				
rs4810485	GG	206(35.64%)	274(45.67%)	**0.0013**	**0.0005**	0.8777	0.1500
	GT	299(51.73%)	252(42.00%)				
	TT	73(12.63%)	74(12.33%)				
rs3765459	GG	258(45.10%)	286(48.89%)	**0.0279**	0.1973	**0.0082**	**0.0081**
	AG	235(41.08%)	247(42.22%)				
	AA	79(13.81%)	52(8.89%)				

Significant values (P<0.05) are in bold.

As shown in Table 1 and Table 2, the SNPs genotyped in this study showed a statistically significant association with breast cancer under different genetic models. For the rs1800686 polymorphism, we observed a higher prevalence of A alleles (P = 0.0275, OR = 1.215, 95%CI [1.022, 1.445]) in breast cancer patients than in controls. Statistical significance was also found using the additive genetic model (GG vs. GA vs. AA, P = 0.0223), the recessive genetic model (AA vs. GG+GA, P = 0.0060) and the homozygote comparison (AA vs. GG, P = 0.0071). Strong associations with breast cancer risk were found for rs1883832 and rs4810485 respectively in the additive genetic model (CC vs. CT vs. TT, P = 0.0012; GG vs. GT vs. TT, P = 0.0013, respectively) and in the dominant genetic model (TT+CT vs. CC, P = 0.0004, TT+GT vs. GG, P = 0.0005, respectively). For the rs1883832 and rs4810485 polymorphisms, different distributions were also observed for the rs1883832 T allele and the rs4810485 T allele between patients and controls, even after correcting the P value for multiple testing using Haploview with 10,000 permutations (P = 0.0193, OR = 1.264 95%CI [1.066,1.499]; P = 0.0250, OR = 1.252 95%CI [1.057,1.482], respectively). Further, in the analysis of rs3765459, the A allele had a higher prevalence among patients (P = 0.0250, OR = 1.221, 95%CI [1.025, 1.454]), and a genotype analysis using different genetic models (additive, GG vs. GA vs. AA, P = 0.0279; recessive, AA vs. GG+GA, P = 0.0082; homozygote comparison, AA vs. GG, P = 0.0081) also indicated a significant association between this SNP and breast cancer risk.

**Table 2:** Alleles of the four SNPs in CD40 gene.

Reference SNP ID	Allele	Frequency No. (%)		P value	OR (95% CI)
		Patients (n = 591)	Controls (n = 600)		
rs1800686	G	764(65.86%)	837(70.10%)		
A/G	A	396(34.14%)	357(29.90%)	**0.0275**	1.215 (1.022,1.445)
rs1883832	C	723(62.22%)	789(67.55%)		
C/T	T	439(37.78%)	379(32.45%)	**0.0070** a	1.264 (1.066,1.499)
rs4810485	G	711(61.51%)	800(75.00%)		
G/T	T	445(38.49%)	400(25.00%)	**0.0090** b	1.252 (1.057,1.482)
rs3765459	G	751(65.65%)	819(70.00%)		
A/G	A	393(34.35%)	351(30.00%)	**0.0250**	1.221(1.025,1.454)

Significant values (p<0.05) are in bold.

a P = 0.0193,

b P = 0.0250 after correcting P value for multiple testing by Haploview program using 10,000 permutations.

Abbreviations: OR, odds ratio; CI, confidence interval.

### Haplotype analysis

As shown in Figure S1, we found that rs1883832 and rs4810485 (D′ = 0.94, R^2^ = 0.87) belonged to the only LD block identified using the Haploview program based on the Solid Spine of LD method. Table 3 lists the haplotypes with frequencies ≧1%. Two of the SNPs (rs1883832 and rs4810485) genotyped were within the only defined haplotype block, which was significantly associated with sporadic breast cancer risk (details in Table 3).

**Table 3:** CD40 haplotype frequencies in breast cancer patients and controls.

Reference SNP ID		Haplotype Frequency	Haplotype Frequency		P value
rs1883832	rs4810485		Patients	Controls	
C	G	0.630	60.48%	65.53%	**0.0107** a
T	T	0.340	36.40%	31.67%	**0.0150** b
C	T	0.017	1.79%	1.67%	0.8131
T	G	0.012	1.33%	1.13%	0.6555

Significant values (P<0.05) are in bold.

a P = 0.0337,

b P = 0.0430 after correcting P value for multiple testing by Haploview using 10,000 permutations.

### Clinical features

The clinical features of the 591 breast cancer patients are summarized in Table 4. The correlation between polymorphisms of CD40 and a series of clinicopathologic features, including histological grade, tumor size, lymph node metastasis and the statuses of ER, PR, C-erbB2 and P53 were analyzed in this study. Detailed results of the statistically significant associations are presented in Table S1, Table S2, Table S3, Table S4 and Table S5 respectively in the Supporting Information section. The most significant associations were observed in analysis of the associated between rs1800686 and the ER and PR statuses. Following correction of the P value for multiple testing using Haploview, the rs1800686 G allele remained statistically more frequent in ER/PR positive cases (ER, P = 0.0015; PR, P = 0.0027, respectively). In the breast cancer patient group, the rs1883832 T allele seemed to provide a protective effect against lymph node metastasis (P = 0.0479), and a statistically significant association was found using the recessive model (TT vs. CC+CT, P = 0.0458) and the homozygote comparison (TT vs. CC, P = 0.0282). We further identified the association between the four SNPs genotyped and the C-erbB2 statuses of breast cancer patients. As shown in Table S4, rs1800686 and rs3765459 were associated with the C-erbB2 statuses of breast cancer patients. Breast cancer patients with the rs1800686 A allele or the rs3765459 A allele had an increased risk of a C-erbB2 positive status compared with patients with the rs1800686 A allele or the rs3765459 A allele (P = 0.0249 and P = 0.0361, respectively). Table S5 presents the relationship between SNPs and the P53 status, and a statistically significant association was found in the rs1800686 allelic P value (P = 0.0223), the rs1800686 recessive genetic model (AA vs. GA+GG, P = 0.0423), the rs1800686 homozygote comparison (AA vs. GG, P = 0.0247), the rs3765459 recessive genetic model (AA vs. GA+GG, P = 0.0491) and the rs3765459 homozygote comparison (AA vs. GG, P = 0.0428). However, no relationships were found for tumor size and histological grade. Overall, these date indicate that rs1800686 and rs3765459 polymorphisms are associated with the ER, PR, C-erbB2 and P53 statuses in breast cancer patients, whereas the rs1883832 and rs4810485 polymorphisms may be involved in breast cancer lymph node metastasis.

**Table 4:** Clinicopathologic information of breast cancer patients (n = 591).

Clinicopathologic information	Case, No. (%)
Tumor Type	
IDC	510(86.29)
MC	13(2.20)
Intraductal carcinoma	31(5.25)
Mucinous adenocarcinoma	4(0.68)
Others	33(5.58)
Tumor Size	
With the diameter less than 2 cm	205(34.69)
With the diameter of 2 to 5 cm	266(45.01)
With the diameter more than 5 cm	35(5.92)
Unknown	85(14.38)
LN involvement	
Positive	259(43.82)
Negative	322(54.48)
Unknown	10(1.69)
ER	
Positive	288(48.73)
Negative	210(35.53)
Unknown	93(15.74)
PR	
Positive	361(61.08)
Negative	135(22.84)
Unknown	95(16.07)
P53	
Positive	116(19.63)
Negative	288(48.73)
Unknown	187(31.64)
C-erbB2	
Positive	156(26.40)
Negative	338(57.19)
Unknown	97(16.41)

Abbreviations: IDC, infiltrative ductal carcinoma; MC, medullary carcinoma; LN, lymph node; ER, estrogen receptor; PR, progesterone receptor; P53, tumor protein 53; C-erbB2, human epidermal growth factor receptor 2.

## Discussion

The CD40 pathway has been recognized as a major component of the anti-tumor response and holds promise as a novel target for therapies for advanced human malignancies [18]. However, accumulating evidence has indicated that CD40 may contribute to tumor proliferation and escape through its special transduction pathway, indicating the multifaceted biological properties of CD40 in cancer [7]. Due to the complex functions of the CD40 pathway in the prognosis of cancer, polymorphisms of the CD40 gene that have crucial roles in the translational efficiency of the CD40 protein may affect the risk and prognosis of breast cancer in Chinese Han women. Four SNPs (rs1800686, rs1883832, rs4810485 and rs3765459) were included in our case-control study. And our results indicate that some of the alleles, genotypes and haplotypes of the CD40 gene are associated with the risk and the clinicopathological features of breast cancer.

In this study, we observed that the rs1800686 AA genotype may increase the risk of breast cancer. The rs1800686 SNP is located at the 5′ near gene region of the CD40 gene, where mutations can modulate CD40 promoter activity. Thus, it is probable that the rs1800686 AA genotype contributes to breast carcinogenesis by lowing CD40 levels on immune cells to suppress the anti-tumor responses. The frequencies of the rs1883832 C allele and the rs4810485 G allele were lower in patients than in controls, even after correcting the P value for multiple testing, suggesting that these two alleles play a protective role in breast cancer. As we know, rs1883832 coincides into the Kozak consensus sequence [19]. Previous functional studies have revealed that individuals with the rs1883832 TT genotype have lower circulating soluble CD40 levels and reduced levels of CD40 on the surfaces of monocyte-derived activated dendritic cells and B cells [15], [16]. Moreover, the rs1883832 CC genotype which could enhance CD40 translational efficiency has been shown to induce the development of autoimmune diseases, such as Grave's disease [16]. The rs4810485 SNP, which is located at intron 1, can play an important role in the splicing processes to regulate CD40 gene expression. It is probable that the rs1883832 T allele located at the Kozak consensus and that the rs4810485 T allele is located at intron 1, which may reduce CD40 expression on immune cell surfaces by hindering the stabilization of the mRNA-ribosome complex [14] and by inducing aberrant splicing, respectively, preventing immune cells from finding and eliminating precancerous cells, thus increasing the breast cancer risk. A similar result was also found for rs3765459, located in intron 8, where mutations may also induce aberrant splicing due to the disruption of the splice site.

In the clinical features analysis, we found that CD40 polymorphisms were associated the clinicopathological features of the patients. For rs1800686 and rs3765459, statistically significant differences were found for PR-positive, ER-positive, C-erbB2-positive cases compared with the negative controls. Steroid hormone receptors influence the disease-free and overall survival of breast cancer patients and are considered to be predictive markers of endocrine therapy [20]–[22]. The expression of C-erbB2 is associated with the tumor metastasis [23], [24]. Thus, the genotypes of rs1800686 and rs3765459 may be good markers to predict the prognosis of breast cancer and the effectiveness of pharmaceutical treatment. Interestingly, although the frequencies of the rs1883832 T and rs4810485 T alleles were found to be higher among breast cancer patients, the frequencies were lower among patients with lymph node involvement. These results indicate that the rs1883832 T and rs4810485 T alleles were risk factors for breast cancer occurrence but protective factors for lymph node metastasis.

The linkage disequilibrium (LD) among the four CD40 polymorphic loci was assessed using Haploview. One LD block including rs1883832 and rs4810485 was identified, which was significantly associated with sporadic breast cancer risk. The haplotype C_rs1883832_G_rs4810485_ was a protective haplotype, whereas T_rs1883832_T_rs4810485_ increased the breast cancer risk in our population, even after correcting the P value for multiple testing (P = 0.0337 and 0.0430, respectively). These haplotypes may also be meaningful in the pathology of breast cancer in our population.

We investigated the association between CD40 gene polymorphisms and the sporadic breast cancer. CD40 gene polymorphisms appear to contribute to sporadic breast cancer risk and had a significant association with clinicopathological features among Chinese Han women from northeast China. However, larger epidemiological studies with ethnically diverse populations as well as the basic functions of CD40 gene mutations need to be further conducted.

## Materials and Methods

### Human specimen collection

Peripheral blood samples were obtained from 591 patients referred at the Department of Abdominal Surgery. (the Third Affiliated Hospital of Harbin Medical University, Heilongjiang Province). The 591 patients consisted in our study are ranged from 20 to 78 years old (mean age at 49.8±7.4 years), who were all diagnosed by surgical and pathological symptoms. Each patient's pathological information was obtained from her medical files. 600 healthy controls were recruited randomly from community volunteers. All of the healthy controls were frequency matched to the patients by age (mean age at 46.3±8.4 years) and did not have a history of personal or familial malignancy or autoimmune disorders. The patients were recruited from 2005 to 2010, and the healthy controls were recruited from 2005 to 2009. This study was conducted in the Heilongjiang Province in northeastern China. This research has the approval instruments of the institutional ethical committees of the Third Affiliated Hospital of Harbin Medical University and Harbin Medical University. All of the volunteers gave written confirmed consent.

### DNA extraction and genotyping

5 ml frozen whole blood was taken to extract the genomic DNA using the Universal Genomic DNA Extraction Kit, version 3.0 (TaKaRa, Japan) according to the manufacturer's protocol. All the genotyping was performed using the polymerase chain reaction restriction fragment length polymorphism (PCR-RFLP) assay. The primers and PCR programs for CD40 PCR-RFLP genotyping are shown in Table S6. The PCR reaction mixture contained 0.3 µg genomic DNA, 1×PCR buffer (Mg^2+^ Free), 0.3 mM MgCl_2_, 0.2 mM dNTPs, 2 U Taq DNA polymerase (Takara, Japan), 0.1 µmol of each primer (Shenggong, China) and ddH_2_O in a final volume of 25 µL. The regions containing polymorphisms were amplified by PCR with a T-Gradient Thermoblock PCR System (BioRad, USA). The restriction enzymes for each SNP were BssSI (rs1800686), NcoI (rs1883832), MspI (rs4810485) and PflmI (rs3765459). For rs1800686/rs3765459, 5 µL of PCR-amplified product was incubated at 37°C for 6–8 h with 0.5 µL of restriction enzyme (BssSI 4000 U/mL, PflmI 8000 U/mL, NEB, USA), 50 mM Tris-HCl, 100 mM NaCl, 10 mM MgCl_2_, 1 mM dithiothreitol, 0.1% BSA and ddH_2_O in a final volume of 10 µL. For rs1883832, 4 µL of PCR-amplified product was incubated at 37°C for 6–8 h with 0.5 µL of restriction enzyme (NcoI 10,000 U/ml, NEB, USA), 50 mM Tris-HCl, 100 mM NaCl, 10 mM MgCl_2_, 1 mM dithiothreitol, and ddH_2_O in a final volume of 10 µL. For rs4810485, 4 µL of PCR-amplified product was incubated at 37°C for 6–8 h with 0.5 µL of restriction enzyme (MspI 20,000 U/ml, NEB, USA), 20 mM Tris-acetate, 50 mM postassium acetate, 10 mM magnesium acetate, 1 mM dithiothreitol, and ddH_2_O in a final volume of 10 µL. The digested fragment lengths for each SNP were as follows: rs1800686 (G: 72+378 bp, A: 450 bp), rs1883832 (C: 61+221 bp, T: 282 bp), rs4810485 (G: 18+148+112 bp, T: 278 bp) and rs3765459 (A: 265+156 bp, G: 421 bp). The results of the 3% agarose gel electrophoresis were analyzed using a Gel Imaging Analysis System and TV lens (Computar, Japan). After PCR-RFLP analysis, purified PCR products of 10% of samples were sequenced directly using an ABI-3730xp automatic DNA sequencer (Applied Biosystems).

### Statistical analysis

The deviation from Hardy-Weinberg equilibrium was determined using a goodness-of-fit Chi-square test to compare the observed genotype frequencies with the expected frequencies among the healthy controls. The polymorphisms were excluded if they deviated from HWE if the minor allele frequency less than 10% or if the missing data comprised more than 10% of the total data. The genotype frequencies of the subjects determined using different genetic models (additive, dominant, recessive and homozygote comparison) was analyzed using the Chi-square test. Haploview was used to infer the haplotype and allele frequencies based on the observed genotypes. To determine the significance corrected for the multiple testing bias, we ran 10,000 permutations to determine the P value using Haploview. All data were analyzed with SPSS (Version 17.0; SPSS, Chicago, Illinois) and Haploview (version 4.1) (http://www.broad.mit.edu/mpg/haploview/). The threshold for significance was P<0.05, and the relative risks associated with rare alleles, genotypes and haplotypes were estimated as odds ratios (ORs) with 95% confidence intervals (CIs).

## Supporting Information

Figure S1 Linkage disequilibrium (LD) block defined by the Haploview program (Linkage disequilibrium block defined by the Haploview program based on the Solid Spine of LD method. Pairwise LD coefficients D′×100 are shown in each cell. The standard color scheme was applied for LD color display.)
(TIF)

Table S1 Significant associations between CD40 SNPs and ER status in patients.
(DOC)

Table S2 Significant associations between CD40 SNPs and PR status in patients.
(DOC)

Table S3 Significant associations between CD40 SNPs and LN involvement status in patients.
(DOC)

Table S4 Significant associations between CD40 SNPs and C-erbB2 status in patients.
(DOC)

Table S5 Significant associations between CD40 SNPs and P53 status in patients.
(DOC)

Table S6 Primers, PCR programs, restriction enzyme and restriction fragments for CD40 PCR-RFLP genotyping.
(DOC)
