# Supplementary figures and images for: Association of CD40 Gene Polymorphisms with Sporadic Breast Cancer in Chinese Han Women of Northeast China
Source: PLoS One. 2011 Aug 30;6(8):e23762. doi: 10.1371/journal.pone.0023762 (PMC3166053; doi:10.1371/journal.pone.0023762)

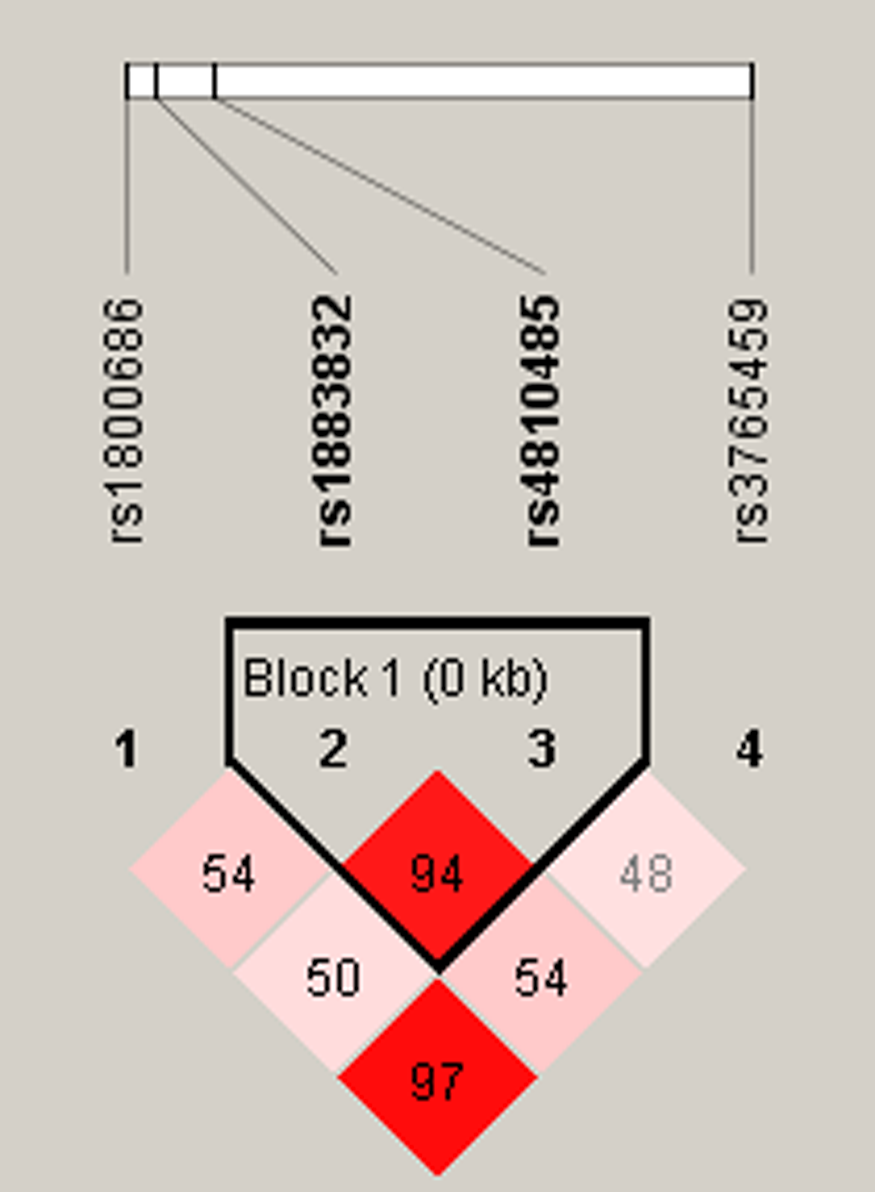

Supplement: Figure S1 — Linkage disequilibrium (LD) block defined by the Haploview program (Linkage disequilibrium block defined by the Haploview program based on the Solid Spine of LD method. Pairwise LD coefficients D′×100 are shown in each cell. The standard color scheme was applied for LD color display.) (TIF) [file pone.0023762.s001.tif]
