# Supplementary material for: Association of CD40 Gene Polymorphisms with Sporadic Breast Cancer in Chinese Han Women of Northeast China
Source: PLoS One. 2011 Aug 30;6(8):e23762. doi: 10.1371/journal.pone.0023762 (PMC3166053; doi:10.1371/journal.pone.0023762)
Supplement: Table S1 — Significant associations between CD40 SNPs and ER status in patients. (DOC) [file pone.0023762.s002.doc]

**Table S1.** Significant associations between CD40 SNPs and ER status in patients

| Reference SNP ID | Genotype | ER status | | Allele | ER status | | Additive P value | Dominant P value | Recessive P value | Homozygote comparison P value | Allelic P value |
| --- | --- | --- | --- | --- | --- | --- | --- | --- | --- | --- | --- |
|  |  | Positive | Negative |  | Positive | Negative |  |  |  |  |  |
| rs1800686 | GG | 138(48.59%) | 74(36.10%) | G | 389(68.49%) | 236(57.56%) | **0.0036** | **0.0059** | **0.0048** | **0.0010** | **0.0004^a^** |
|  | AG | 113(39.79%) | 88(42.93%) | A | 179(31.51%) | 174(42.44%) |  |  |  |  |  |
|  | AA | 33(11.62%) | 43(20.98%) |  |  |  |  |  |  |  |  |
| rs3765459 | GG | 130(46.76%) | 77(38.12%) | G | 372(66.91%) | 242(59.90%) | 0.1031 | 0.0591 | 0.1060 | **0.0437** | **0.0256** |
|  | AG | 112(40.29%) | 88(43.56%) | A | 184(33.10%) | 162(40.10%) |  |  |  |  |  |
|  | AA | 36(12.95%) | 37(18.32%) |  |  |  |  |  |  |  |  |

*Significant values (P<0.05) are in bold.

^a^P=0.0015 after correcting P value for multiple testing by Haploview using 10,000 permutations.

Abbreviation: ER, estrogen receptor.
