# Supplementary material for: Association of CD40 Gene Polymorphisms with Sporadic Breast Cancer in Chinese Han Women of Northeast China
Source: PLoS One. 2011 Aug 30;6(8):e23762. doi: 10.1371/journal.pone.0023762 (PMC3166053; doi:10.1371/journal.pone.0023762)
Supplement: Table S2 — Significant associations between CD40 SNPs and PR status in patients. (DOC) [file pone.0023762.s003.doc]

**Table S2.** Significant associations between CD40 SNPs and PR status in patients

| Reference SNP ID | Genotype | PR status | | Allele | PR status | | Additive P value | Dominant P value | Recessive P value | Homozygote comparison P value | Allelic P value |
| --- | --- | --- | --- | --- | --- | --- | --- | --- | --- | --- | --- |
|  |  | Positive | Negative |  | Positive | Negative |  |  |  |  |  |
| rs1800686 | GG | 163(45.92%) | 47(35.61%) | G | 475(66.90%) | 146(55.30%) | **0.0017** | **0.0412** | **0.0005** | **0.0005** | **0.0008^a^** |
|  | AG | 149(41.97%) | 52(39.39%) | A | 235(33.10%) | 118(44.70%) |  |  |  |  |  |
|  | AA | 43(12.11%) | 33(25.00%) |  |  |  |  |  |  |  |  |

*Significant values (p<0.05) are in bold.

^a^P=0.0027 after correcting P value for multiple testing by Haploview using 10,000 permutations.

Abbreviation: PR, progesterone receptor.
