# Supplementary material for: Association of CD40 Gene Polymorphisms with Sporadic Breast Cancer in Chinese Han Women of Northeast China
Source: PLoS One. 2011 Aug 30;6(8):e23762. doi: 10.1371/journal.pone.0023762 (PMC3166053; doi:10.1371/journal.pone.0023762)
Supplement: Table S3 — Significant associations between CD40 SNPs and LN involvement status in patients. (DOC) [file pone.0023762.s004.doc]

**Table S3.** Significant associations between CD40 SNPs and LN involvement status in patients

| Reference SNP ID | Genotype | LN involvement | | Allele | LN involvement | | Additive P value | Dominant P value | Recessive P value | Homozygote comparison P value | Allelic P value |
| --- | --- | --- | --- | --- | --- | --- | --- | --- | --- | --- | --- |
|  |  | Positive | Negative |  | Positive | Negative |  |  |  |  |  |
| rs1883832 | CC | 102(39.84%) | 107(33.97%) | C | 334(65.23%) | 375(59.52%) | 0.0895 | 0.1472 | **0.0458** | **0.0282** | **0.0479** |
|  | CT | 130(50.78%) | 161(51.11%) | T | 178(34.77%) | 255(40.48%) |  |  |  |  |  |
|  | TT | 24(9.38%) | 47(14.92%) |  |  |  |  |  |  |  |  |
| rs4810485 | GG | 97(38.04%) | 106(33.87%) | G | 327(64.12%) | 371(59.27%) | 0.1300 | 0.3019 | 0.0501 | **0.0458** | 0.0947 |
|  | GT | 133(52.16%) | 159(50.80%) | T | 183(35.88%) | 255(40.73%) |  |  |  |  |  |
|  | TT | 25(9.80%) | 48(15.34%) |  |  |  |  |  |  |  |  |

*Significant values (P<0.05) are in bold.

Abbreviation: LN, lymph node.
