# Supplementary material for: Association of CD40 Gene Polymorphisms with Sporadic Breast Cancer in Chinese Han Women of Northeast China
Source: PLoS One. 2011 Aug 30;6(8):e23762. doi: 10.1371/journal.pone.0023762 (PMC3166053; doi:10.1371/journal.pone.0023762)
Supplement: Table S4 — Significant associations between CD40 SNPs and C-erbB2 status in patients. (DOC) [file pone.0023762.s005.doc]

**Table S4.** Significant associations between CD40 SNPs and C-erbB2 status in patients

| Reference SNP ID | Genotype | C-erbB2 status | | Allele | C-erbB2 status | | Additive P value | Dominant P value | Recessive P value | Homozygote comparison P value | Allelic P value |
| --- | --- | --- | --- | --- | --- | --- | --- | --- | --- | --- | --- |
|  |  | Positive | Negative |  | Positive | Negative |  |  |  |  |  |
| rs1800686 | GG | 48(32.21%) | 162(48.21%) | G | 175(58.72%) | 445(66.22%) | **0.0012** | **0.0010** | 0.7768 | 0.2634 | **0.0249** |
|  | AG | 79(53.02%) | 121(36.01%) | A | 123(41.28%) | 227(33.78%) |  |  |  |  |  |
|  | AA | 22(14.77%) | 53(15.77%) |  |  |  |  |  |  |  |  |
| rs3765459 | GG | 49(32.24%) | 156(48.15%) | G | 180(59.21%) | 429(66.20%) | **0.0008** | **0.0011** | 0.5847 | 0.3766 | **0.0361** |
|  | AG | 82(53.95%) | 117(36.11%) | A | 124(40.79%) | 219(33.80%) |  |  |  |  |  |
|  | AA | 21(13.82%) | 51(15.74%) |  |  |  |  |  |  |  |  |

*Significant values (P<0.05) are in bold.

Abbreviation: C-erbB2, human epidermal growth factor receptor 2.
