# Supplementary material for: Association of CD40 Gene Polymorphisms with Sporadic Breast Cancer in Chinese Han Women of Northeast China
Source: PLoS One. 2011 Aug 30;6(8):e23762. doi: 10.1371/journal.pone.0023762 (PMC3166053; doi:10.1371/journal.pone.0023762)
Supplement: Table S5 — Significant associations between CD40 SNPs and P53 status in patients. (DOC) [file pone.0023762.s006.doc]

**Table S5.** Significant associations between CD40 SNPs and P53 status in patients

| Reference SNP ID | Genotype | P53 status | | Allele | P53 status | | Additive P value | Dominant P value | Recessive P value | Homozygote comparison P value | Allelic P value |
| --- | --- | --- | --- | --- | --- | --- | --- | --- | --- | --- | --- |
|  |  | Positive | Negative |  | Positive | Negative |  |  |  |  |  |
| rs1800686 | GG | 42(37.84%) | 134(47.02%) | G | 130(58.56%) | 383(67.19%) | 0.0802 | 0.0987 | **0.0423** | **0.0247** | **0.0223** |
|  | AG | 46(41.44%) | 115(40.35%) | A | 92(41.44%) | 187(32.81%) |  |  |  |  |  |
|  | AA | 23(20.72%) | 36(12.63%) |  |  |  |  |  |  |  |  |
| rs3765459 | GG | 43(39.09%) | 128(46.04%) | G | 130(59.09%) | 370(66.55%) | 0.1245 | 0.2138 | **0.0491** | **0.0428** | 0.0505 |
|  | AG | 44(40.00%) | 114(41.01%) | A | 90(40.91%) | 186(33.45%) |  |  |  |  |  |
|  | AA | 23(20.91%) | 36(12.95%) |  |  |  |  |  |  |  |  |

*Significant values (P<0.05) are in bold.

Abbreviation: P53, tumor protein 53.
