# Supplementary material for: Association of CD40 Gene Polymorphisms with Sporadic Breast Cancer in Chinese Han Women of Northeast China
Source: PLoS One. 2011 Aug 30;6(8):e23762. doi: 10.1371/journal.pone.0023762 (PMC3166053; doi:10.1371/journal.pone.0023762)
Supplement: Table S6 — Primers, PCR programs, restriction enzyme and restriction fragments for CD40 PCR-RFLP genotyping. (DOC) [file pone.0023762.s007.doc]

**Table S6.** Primers, PCR programs, restriction enzyme and restriction fragments for CD40 PCR-RFLP genotyping

| Reference SNP ID | Gene/SNP primer sequence | PCR program | Assay | Restriction enzyme | Restriction fragments |
| --- | --- | --- | --- | --- | --- |
| rs1800686 | F:5'-GATGGAATGGAATGAGGTG-3' | 94°C 5min, 30cycles, 94°C 30s, 54°C 30s,72°C 30s, 72°C 5min | PCR-  RFLP | BssSI | G allele: 72 bp+378 bp, A allele: 450 bp |
| A/G | R:5'-AGTTTAGGGACGCATCTTG-3' |
| rs1883832 | F:5'-AAGATGCGTCCCTAAACTC-3' | 94°C 5min, 30cycles, 94°C 30s, 55.4°C 30s,72°C 30s, 72°C 5min | PCR-  RFLP | NcoI | C allele: 61 bp+221 bp, T allele: 282 bp |
| C/T | R:5'-ACAACTCACAGCGGTCAG-3' |
| rs4810485 | F:5'-GCCAACCAAGCATATCTC-3' | 94°C 5min, 30cycles, 94°C 30s, 55°C 30s,72°C 30s, 72°C 5min | PCR-  RFLP | MspI | G allele: 18 bp+148 bp+112 bp, T allele: 278 bp |
| G/T | R:5'-GGTGAAAGTGAAAGCTGTG-3' |
| rs3765459 | F:5’-ATGCTCCTTCCATCCAGA-3’ | 94°C 5min, 30cycles, 94°C 30s, 54°C 30s,72°C 30s, 72°C 5min | PCR-  RFLP | PflmI | A allele: 265 bp+156 bp, G allele: 421 bp |
| A/G | R:5’-TCGTCGGGAAAATTGATCTCCT-3’ |

Abbreviations: PCR-RFLP, polymerase chain reaction restriction fragment length polymorphism; F, forward primer; R, reverse primer.
